# The effects of gain-loss framed message on physical activity attitudes, intentions, and behaviors in physically inactive adults: a systematic review and meta-analysis

**DOI:** 10.3389/fpubh.2026.1782478

**Published:** 2026-03-16

**Authors:** Yueying Jiang, Yuan Zhao, Qunyan Xu, Panpan Tang, Xueqing Wang, Yunyu Guo, Yue Zhao, Jing Shao, Leiwen Tang

**Affiliations:** 1 Department of Nursing, The Second Affiliated Hospital of Zhejiang University School of Medicine, Hangzhou, China; 2 Adjunct Research Academic, Clinical and Health Sciences, University of South Australia, Adelaide, SA, Australia; 3 School of Nursing, Zhejiang University School of Medicine, Zhejiang University, Hangzhou, China

**Keywords:** physical activity, message framing, gain-framed message, loss-framed message, systematic review, meta-analysis, physically inactive adults

## Abstract

**Background:**

Physical inactivity poses serious health risks. Message framing is a strategy to encourage healthy behaviors, but its effectiveness in promoting physical activity (PA) is unclear, creating challenges for professionals in choosing optimal strategies.

**Objective:**

To compare the effects of gain-framed versus loss-framed messages on PA attitudes, intentions, and behaviors among inactive adults.

**Methods:**

Seven electronic databases were searched from inception to December 15, 2024. Randomized controlled trials examining gain- versus loss-framed messages targeting physically inactive adults were included. Independent reviewers selected the studies, extracted the data, and performed the risk of bias. Standardized mean differences (SMDs) and 95% confidence intervals (CIs) werepooled using random-effects models. Heterogeneity was assessed using *I*^2^ and Q statistics. Subgroup and sensitivity analyses were conducted to explore heterogeneity sources and assess result stability.

**Results:**

Ten RCTs involving 1,355 participants were included. Three articles tested the gain-loss framing effect through PA attitudes, and eight studies each tested message framing through PA intentions and behaviors. There were no statistically significant differences between gain-framed and loss-framed messages in promoting PA attitudes [SMD:−0.60, 95%CI: (-2.09, 0.90), *P* = 0.44], intentions [SMD: 0.10, 95%CI: (-0.12, 0.33), *P* = 0.37] and behaviors [SMD: 0.15, 95%CI: (-0.04, 0.34), *P* = 0.13]. Subgroup analyses suggested that gain-framed messages may have a potential advantage in promoting PA behaviors among general populations [SMD: 0.39, 95%CI: (0.03, 0.55), *P* = 0.03] and young adults [SMD: 0.38, 95%CI: (013, 0.63), *P* = 0.003].

**Conclusions:**

Current evidence does not demonstrate a clear overall advantage of gain- over loss-framed messages in promoting PA among inactive adults. However, gain-framed messages may be more promising for general and young populations. Further high-quality trials with standardized methodologies and longer follow-up periods are needed to clarify their effectiveness.

**Systematic review registration:**

https://www.crd.york.ac.uk/prospero/display_record.php?ID=CRD42023482474, identifier CRD42023482474.

## 1 Introduction

Physical inactivity is a major global public health concern and is associated with an increased the risk of non-communicable illnesses and all-cause mortality. It is defined as engaging in less than 150 min of moderate-to-vigorous physical activity (PA) per week, according to the recommendations of World Health Organization (WHO) (1). Since 2001, global PA levels has shown little improvement, with more than one quarter of the global adult population (1.4 billion adults) failing to meet the WHO guideline for PA (2). The WHO 2020 Guidelines on Physical Activity and Sedentary Behavior emphasize that regular PA confers substantial physical and mental health benefits across the lifespan, including reduced risk of cardiovascular disease, hypertension, diabetes, cancer, and the onset of dementia (3–5). Epidemiological evidence indicates that physically inactive individuals have a 20% to 30% higher risk of all-cause mortality compared with their physically active counterparts. Therefore, effective interventions are needed to promote regular participation in PA.

Effective message design and delivery are critical to the successful promotion of PA, as they can enhance the dissemination of PA guidelines and related recommendations (6, 7). This is particularly important for physically inactive individuals, who demonstrate greater potential for improvement and may be more responsive to PA-related messages (8). Message framing refers to a communication strategy that alters how information is presented in order to influence decision-making, without changing the substantive content of the message (9). It offers a new perspective for promoting health behaviors and has been shown to be an effective approach for increasing the persuasiveness of the message (10, 11). In the context of PA promotion, message can be structured as “gain-framed”, which emphasizes the benefits of engaging in a healthy behavior (eg. Exercising regularly can help you lose weight), whereas “loss-framed” emphasizes the negative consequences of failing to engage in the healthy behavior (eg. Not exercising regularly can lead to weight gain) (12). Additionally, some scholars have suggested that message framing studies should also focus on the description of outcomes, which can be described as outcomes attained or avoided as a result of healthy behaviors, such as PA avoiding heart disease, or maintaining a healthy heart (13, 14). The basic state that the message mentions in describing the consequences is known as the kernel state of message (14). Depending on the kernel state, the outcome of behavior can be described as either attaining desirable/undesirable one or avoiding desirable/undesirable one (14). Accordingly, gain- and loss-framed messages can each take two forms.

Message framing has been widely applied to promote a range of healthy behavior, including PA, smoking cessation, and cancer screening (15–19). According to the theoretical framework proposed by Rothman and Salovey, gain-framed messages are generally more effective for low-risk, prevention behaviors such as healthy eating and PA, whereas loss-framed messages are considered more persuasive for high-risk detection and screening behaviors (13, 20). However, there has been controversy regarding the effectiveness of gain-loss framing messages to promote PA among physically inactive individuals. Most previous studies have reported an advantage of gain-framd message in encouraging PA (8, 21–23), suggesting that emphasizing potential gains may be more effective in motivating individuals to adopt health-protective behaviors than focusing on potential losses. Some studies have yielded the opposite results. Bassett-Gunter and Kin-Kit Li revealed that spinal cord injury patients and older adults with diabetes who received loss-framed messages exhibited stronger PA intentions and more positive behavioral outcomes than those who were exposed to gain-framed messages (24, 25). One possible explanation is that individuals with chronic conditions may perceive higher risks associated with physical inactivity, thereby responding more favorably to loss-framed appeals. Furthermore, several primary studies and meta-analysis have found no significant differences between gain-framed messages and loss-framed messages in promoting PA (26–28). These conflicting findings make it challenging for health professionals and researchers to determine the most effective message framing strategy for physically inactive individuals.

This review aims to systematically compare the effects of gain- and loss-framed messages on PA attitudes, intentions, and behaviors among physically inactive adults. The findings are intended to provide clinicians and researchers with recommendations for designing effective messaging frames to promote PA.

## 2 Methods

This systematic review was reported according to the Preferred Reporting Items for Systematic Reviews and Meta-Analyses (PRISMA) guidelines (29), and its protocol was registered to the International Prospective Register of Systematic Review (PROSPERO) with the registration number CRD42023482474.

### 2.1 Data source and searches

A comprehensive literature search was performed in PubMed, Embase, Web of Science, Cochrane Library, Cumulative Index to Nursing and Allied Health Literature (CINAHL), China National Knowledge Infrastructure (CNKI), and Wanfang Data Knowledge Service Platform from database inception to 15 December 2024. The search strategy combined Medical Subject Headings (MeSH) and free-text using Boolean Logic operators “AND” and “OR”. The core search concepts included *physical activity, message framing, attitude, intention*, and *behavior*. A second search was then conducted based on the reference lists of identified studies and articles to ensure the comprehensiveness of the search. The detailed search strategy can be found in Supplementary 1.

### 2.2 Eligibility criteria

Studies were included if they met the following criteria: (1) Adults aged 18 years and over who are not physically active enough to meet the WHO physical activity guideline (i.e., engaging in fewer than 150 min of moderate-to-vigorous physical activity (MVPA) per week); (2) gain-framed and loss-framed PA promotion messages have to be presented in pairs for comparison; (3) PA attitudes, intentions, or behaviors were examples of outcome indicators, which were quantitative data that have been assessed by scales, questionnaires, accelerometers, or pedometers; (4) randomized controlled trials (RCTs).

The exclusion criteria were: (1) articles published in languages other than Chinese or English; (2) studies without full-text access; (3) systematic reviews, gray literature, editorial letters, and conference abstracts.

### 2.3 Data selection and extraction

EndNote X9 software was used to manage references and identify duplicate records. Following the inclusion and exclusion criteria, an initial screening was conducted based on article title and abstract. Full-text articles were subsequently reviewed to determine final eligibility. This process was independently conducted by two team members. In the event of disagreement, a third researcher joined to consult and make a final decision together.

Data extraction was independently conducted by two reviewers using a standardized data extraction form. The following data were extracted: 1) basic study characteristics including the first author, year of publication, and country; 2) participant characteristics including age, population, gender ratio, and sample size; 3) intervention characteristics including message design, message delivery channel, follow-up time; 4) outcome measures, and means (M) and standard deviations (SD) of outcome indicators at the first time point post-intervention.

### 2.4 Risk of bias appraisal

Version 2 of the Cochrane risk-of-bias (RoB 2.0) (30) tool was employed to evaluate the risk of bias of articles in terms of five domains: randomization process, deviations from intended interventions, missing outcome data, measurement of the outcome, and selection of the reported results. Each domain was classified as “low,” “some concerns,” or “high” risk of bias. The overall risk is assessed based on the risk of each domain.

### 2.5 Data synthesis and analysis

The meta-analysis was performed using Review Manager Software. Egger's regression test and sensitivity analysis were conducted using *R* Version 3.5.1. The three measures of attitudes, intentions, and behaviors were used as indicators of message effectiveness in the meta-analysis, all of which are continuous variables. Standardized mean difference (SMDs) calculated between gain-framed (intervention group) and loss-framed (control group) messages determined the effect size due to variations in measurement scales across studies. Statistical heterogeneity was assessed using the Cochran Q test and Higgins' *I*^2^ statistic. A *P*-value < 0.10 for the *Q* test was considered indicative of statistically significant heterogeneity. I^2^ values of 25%, 50%, and 75% were interpreted as representing low, moderate, and high heterogeneity, respectively. Given the expected methodological and clinical diversity across studies (e.g., differences in populations, message delivery formats, and follow-up durations), a random-effects model was prespecified to estimate pooled effects. This approach assumes that the true effect sizes may vary across studies and provides more conservative estimates when between-study variability is present. Subgroup analysis of population, age, follow-up time, and message exposure dosage were conducted to identify the source of heterogeneity for analysis. To assess the robustness and reliability of the pooled results, a sensitivity analysis was conducted using a one-study-out method. Lastly, Egger's regression tests were used to identify publication bias.

### 2.6 Assessment of certainty of evidence

The certainty of the evidence for each primary outcome (attitudes, intentions, and behaviors) was independently evaluated using the Grading of Recommendations Assessment, Development and Evaluation (GRADE) framework (31). Evidence from randomized controlled trials was initially rated as high certainty and subsequently downgraded based on five domains: risk of bias, inconsistency, indirectness, imprecision, and publication bias. The GRADE assessment was conducted independently by two reviewers, with disagreements resolved through discussion.

## 3 Results

### 3.1 Search results

**Figure 1:**
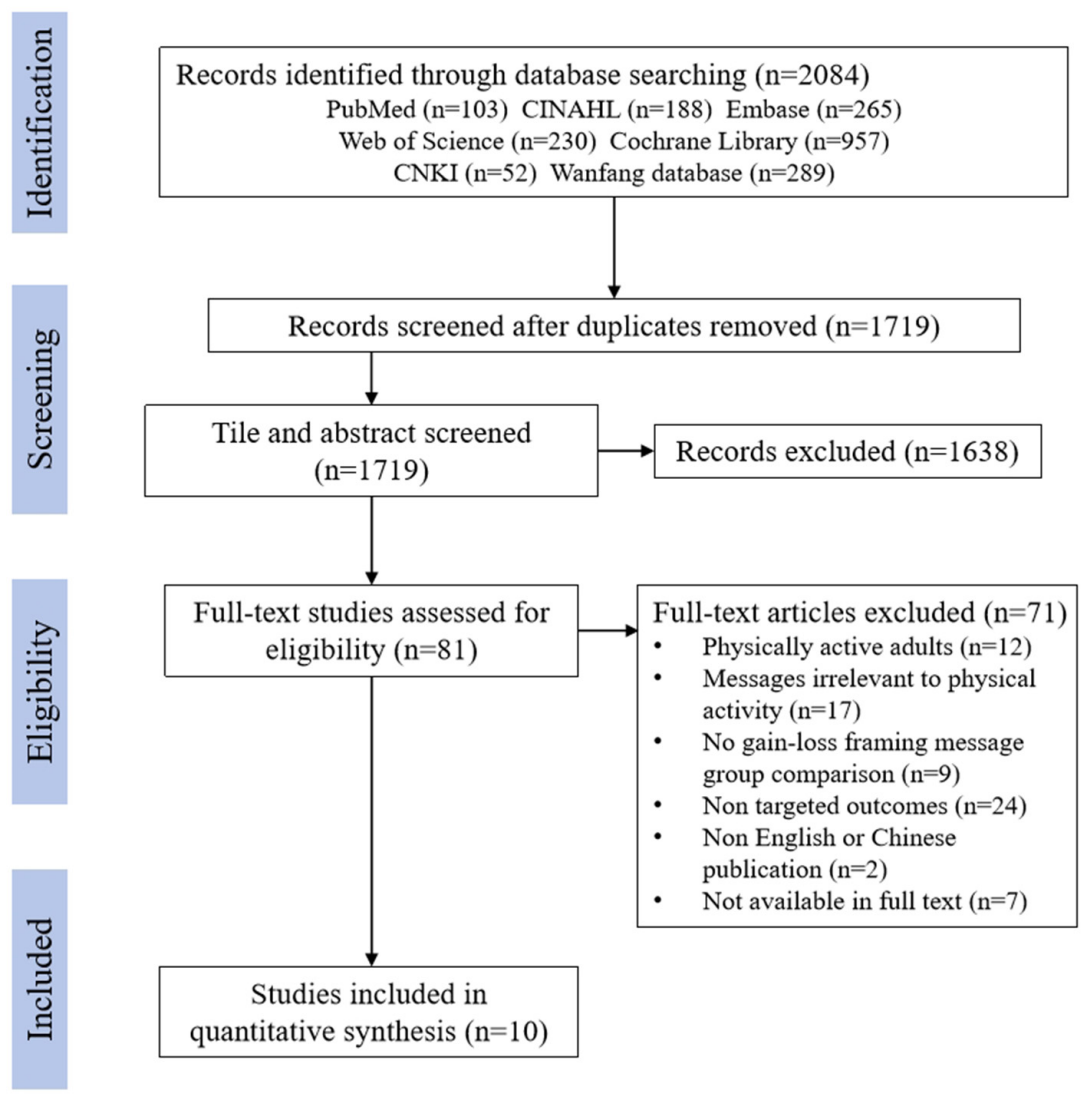
PRISMA flow diagram.

A total of 2,084 articles were initially retrieved. After removing 365 duplicates, the titles and abstracts of 1,719 articles were screened for eligibility. Subsequently, the full text of 81 articles was further assessed. Ultimately, 10 studies were included in the meta-analysis. The search and selection flowchart is presented in Figure 1.

### 3.2 Study characteristics

The characteristics of the included studies are shown in of Supplementary Table 1. These studies conducted in four countries: USA (*n* = 2), Canada (*n* = 4), China (*n* = 3), and Italy (*n* = 1). A total of 1,355 were included in the meta-analysis, with 684 in the intervention group and 671 in the control group. The mean age of participants ranged from 20.10 to 71.66 years, and the proportion of female participants was 39.4–100%. Although our inclusion criteria defined low physical activity as engaging in fewer than 150 min of moderate-to-vigorous physical activity (MVPA) per week, the operational definition of insufficient physical activity varied slightly across the included studies. Specifically, some studies defined low physical activity as engaging in < 150 min of MVPA per week, whereas others used alternative criteria, such as engaging in MVPA on ≤ 2 days per week, failing to achieve 30 min of MVPA on ≥4 days per week, or not completing at least three 20-min bouts of MVPA per week. Detailed inclusion criteria for each study are provided in Supplementary Table 1. Most studies (60%) recruited participants from the general population (8, 21–23, 32, 33), with four studies including patients with spinal cord injury (SCI), multiple sclerosis, Type 2 diabetes, and colorectal cancer (CRC) survivors (24–27).

All message designs incorporated comparisons between gain- and loss-framed PA messages. Three studies further explored the interaction of the kernel state of the message with the gain-loss frame (23, 32, 33) and one explored the interaction of risk messages with it (26). The interventions were delivered through both online and offline channels. Online dissemination methods include leaflets, electronic links, digital print ads, and mobile applications (21, 23, 24, 26, 32). In contrast, offline dissemination strategies were more limited and primarily relied printed materials (8, 22, 25, 27, 33).

Most studies assessed attitudes and intentions immediately following the message intervention (21, 23–26, 32, 33), whereas PA behaviors were typically measured at the 2-week post-intervention (8, 22, 23, 25, 33). Four studies additionally evaluated behavior change indicators at 1 week, 1 month, 9 weeks, and 12 months after the intervention (8, 21, 26, 27). Attitudes and intentions were primarily measured using researcher self-developed questionnaires, and a higher score means more positive attitudes or higher intentions toward PA. The International Physical Activity Questionnaire (IPAQ) is the most commonly used tool for measuring PA behavior (8, 21, 22, 33), followed by the Godin Leisure-Time Exercise Questionnaire (GLTEQ) (27). The study by Lithopoulos et al. (26) utilized the specific Leisure-time PA questionnaire for people with spinal cord injury (LTPAQ-SCI) to measure the PA behavior of SCI patients. Additionally, two studies employed accelerometer measurements (22, 25), and one study utilized physical activity frequency to measure PA behavior (23).

### 3.3 Quality of included studies

**Figure 2:**
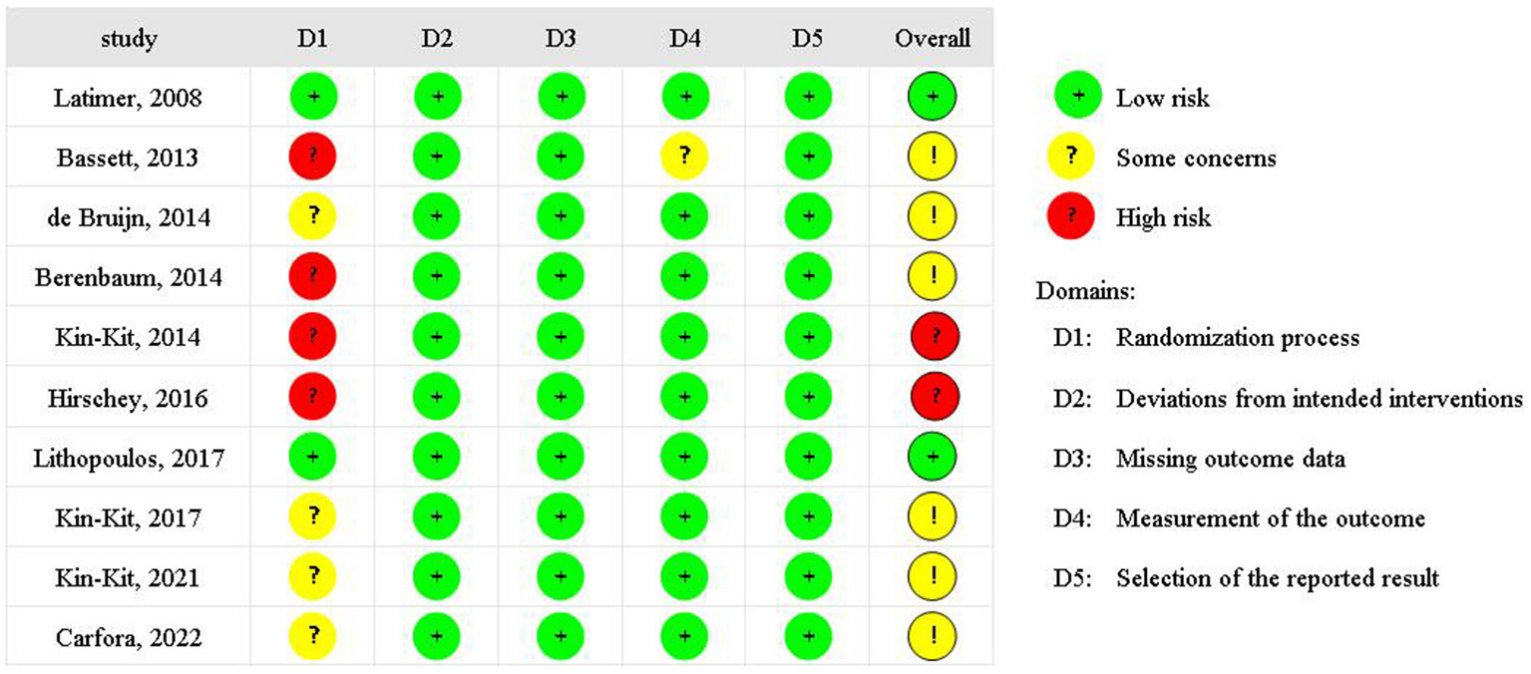
Risk bias of these studies.

As shown in Figure 2, only two studies reported the randomized process methodology and were deemed to have a low risk of bias, four studies presented some concerns and four studies presented a high risk of bias. All studies presented a low risk of deviations from intended interventions, missing outcome data, and measurement selection of the reported result. For bias from measurement of the outcome, one study presented some concerns. Egger's regression test indicated no evidence of publication bias for attitudes (*P* = 0.989), intentions (*P* = 0.317), or behaviors (*P* = 0.104).

### 3.4 Effects of message frame inventions

#### 3.4.1 PA Attitude

**Figure 3:**
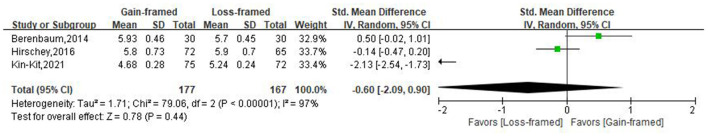
Forest plot of message framing on PA attitudes.

Three studies used attitude to indicate the persuasiveness of PA messages. The relevant data involved a total of 344 participants, including 177 in the gain-framed messaging group and 167 in the loss-framed group. There was no evidence of a difference in PA attitudes between the gain-framed and loss-framed messaging [SMD:−0.60, 95%CI: (-2.09, 0.90), *P* = 0.44] (Figure 3), and sensitivity analysis reflected that no individual trial was able to change the result (Supplementary Figure 8).

#### 3.4.2 PA Intention

**Figure 4:**
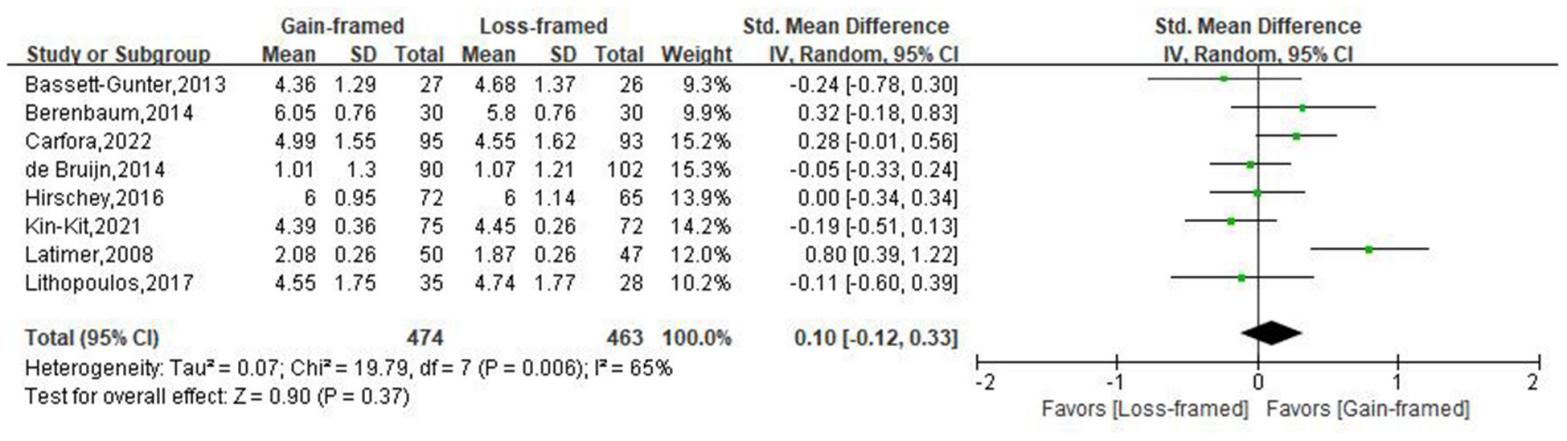
Forest plot of message framing on PA intentions.

Eight studies, involving 937 patients (474 in the gain-framed group, 463 in the loss-framed group), reported intention to access the persuasiveness of PA messages. The findings in Figure 4 indicated that there was no evidence of a difference in PA intentions between gain- and loss-framed group [SMD: 0.10, 95%CI: (-0.12, 0.33), *P* = 0.37]. There was moderate heterogeneity (*I*^2^ = 65%, *P* < 0.05) in these studies. Subgroup analysis based on population, age, and duration of follow-up, message exposure dosage also revealed no evidence of differences and sensitivity analysis confirmed the stability of the results (Supplementary Figures 1–4, 9).

#### 3.4.3 PA Behavior

**Figure 5:**
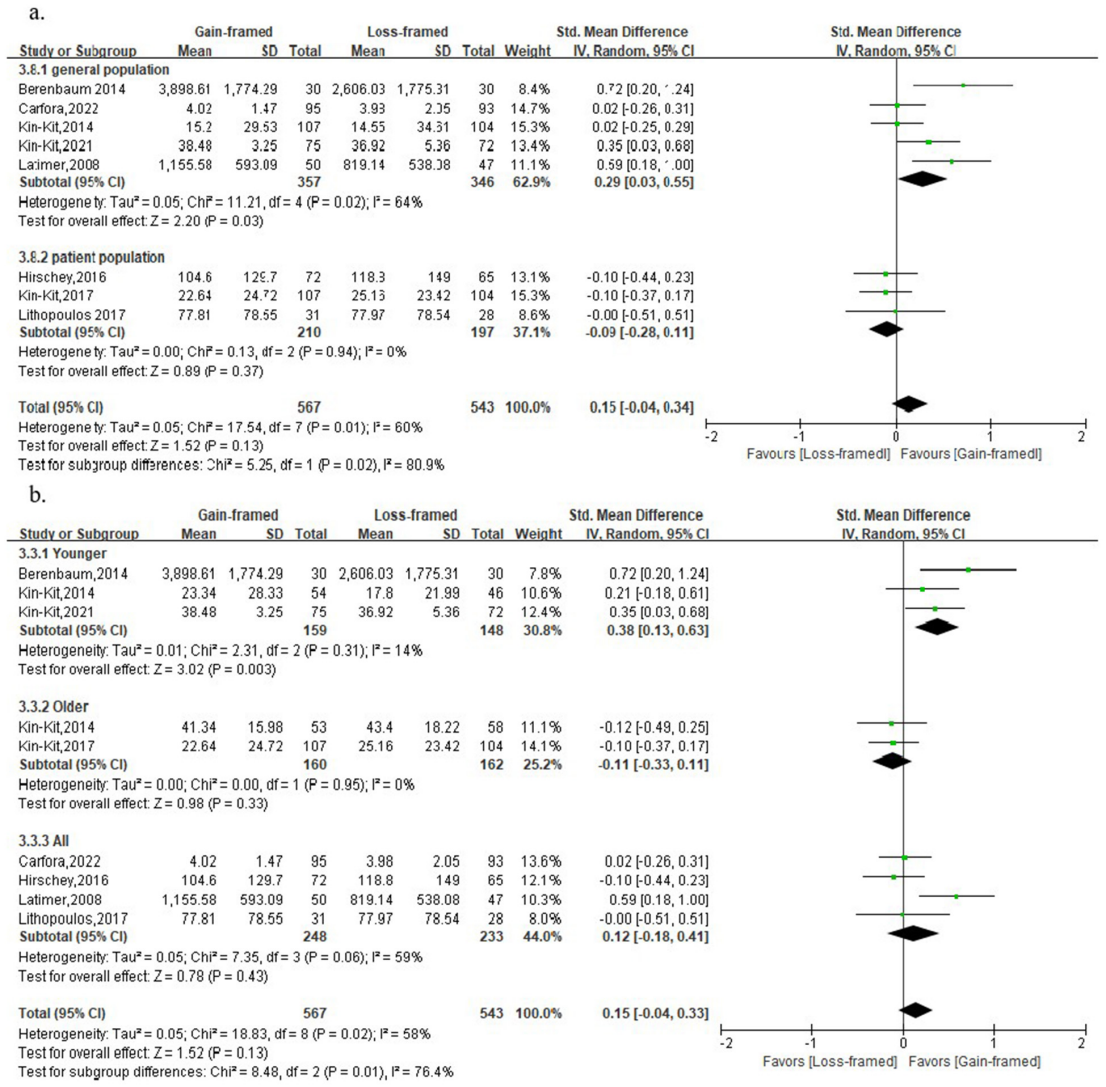
Forest plot of message framing on PA behaviors. **a**. Subgroup analyses of message framing on PA behavior with different healthy statuses. **b**. Subgroup analyses of message framing on PA behavior with different ages. General population: Individuals without specific diseases recruited for the general public, such as adults, college students, and so on. Younger: 18–59 years old; Older: ≥60 years old.

The behavior was used to measure the persuasiveness of messages about PA in 8 studies involving 1,110 participants, with 567 in the gain-framed group and 543 in the loss-framed group. No statistical evidence was found between the two groups [SMD: 0.15, 95%CI: (-0.04, 0.34), *P* = 0.13], with medium heterogeneity (*I*^2^ = 60%, *P* < 0.05) (Supplementary Figure 5). The results of the subgroup analysis of follow-up duration and message exposure dosage also did not show any evidence of differences (Supplementary Figures 6, 7). However, subgroup analysis based on population, and age indicated that gain-framed messages were more effective in promoting PA behaviors in general populations [SMD: 0.39, 95%CI: (0.03, 0.55), *P* < 0.05] (Figure 5) and young adults [SMD: 0.38, 95%CI: (0.13, 0.63), *P* < 0.05] (Figure 5). Sensitivity analysis confirmed the stability of the results (Supplementary Figure 10).

### 3.5 Certainty of evidence

According to the GRADE assessment, the certainty of evidence for attitudes was rated as very low due to very serious inconsistency (*I*^2^ = 97%) and concerns regarding risk of bias. The certainty of evidence for both intentions and behaviors was rated as low. The evidence was downgraded due to moderate heterogeneity (*I*^2^>50%) and methodological limitations in some studies. No serious concerns were identified regarding indirectness or publication bias.

## 4 Discussion

This review analyzed synthesized evidence from10 randomized controlled trials examining the comparative effects of gain- and loss-framed messages on PA attitudes, intentions, and behaviors among physically inactive individuals. Overall, no significant differences were observed between gain- and loss-framed messages across the pooled analyses. However, subgroup findings suggested that gain-framed messages may be more effective in promoting PA behavior among general populations and younger adults. The overall certainty of the evidence ranged from low to very low, and substantial heterogeneity was observed across studies.

The attitudes and intentions toward PA found in this study align with Gallagher and O'Keefe's studies, while our findings on PA behaviors differ from theirs. Their meta-analysis found that no gain-loss message framing effect on PA attitudes and intentions but reported a modest advantage of gain-framed messages in promoting PA behaviors (12, 34). There may be several reasons for this. First, There were important differences in population characteristics and inclusion criteria. Gallagher and O'Keefe included a broader range of health behaviors and populations, whereas our review specifically focused on physically inactive individuals. The effectiveness of gain-framed and loss-framed messages in promoting PA is a complex area, with research indicating that baseline activity levels and various psychological factors can moderate their impact (35, 36). Behavior change among inactive individuals may require stronger motivational activation and higher perceived behavioral control (PBC), which may not be sufficiently influenced by framing alone (37). For inactive individuals, who often exhibit low intention, low PBC, and weak motivational activation, framing effects might be attenuated. The heterogeneity in baseline activity levels among participants in meta-analyses can indeed influence the observed effectiveness of framed appeals. Some studies included participants with more varied baseline activity levels, potentially increasing their responsiveness to gain-framed messages (36). In contrast, restricting our sample to inactive individuals may have reduced variability in readiness for change and limited observable framing effects. Second, intervention design characteristics differed substantially. Earlier research often examined relatively simple gain-versus-loss framing manipulations delivered in more controlled experimental formats (6). In contrast, several trials in our review incorporated additional design elements, such as kernel state variations, mixed framing conditions, or supplementary informational components (e.g., risk statistics). These additions, while potentially enriching the message, can obscure the distinct effects of the primary framing manipulation. The involvement of the issue itself has been shown to moderate the efficacy of gain and loss frames across various behaviors and contexts, indicating that supplementary information or complex framing might interact with an individual's pre-existing involvement with the health issue (38). Differences in message delivery channels and exposure frequency may also have influenced cognitive processing depth and engagement (23). For instance, repeated exposure has been shown to enhance elaborative processing, leading to a deeper understanding and stronger behavioral impact. Third, most studies in our review assessed outcomes immediately or within 2 weeks after a single exposure, which may limit the detection of sustained behavioral effects. Since it has been demonstrated that there is a temporal effect of message framing on PA promotion, and a study by Latimer et al. showed that repeated exposure to PA messages leads to greater behavioral change, because it prompts more deliberate and in-depth processing of the information (8, 23, 26, 39). Finally, the RCTs included in this review had a relatively high risk of bias during the randomization process, which led to an imbalance in the population of these studies at baseline, with some impact on the results. In addition, the small sample size also contributed to the absence of statistical differences. Therefore, more rigorous study designs should be conducted in larger populations with increased frequency and duration of interventions to explore in depth the mechanisms of gain-loss framing messages in PA promotion in inactive individuals in the future.

The preliminary findings of our study suggest that the evidence supporting a gain-framed advantage for PA behaviors is primarily observed in inactive general populations and young adults, which differs from the findings of McCall et al. and Liu et al. McCall et al. reported that gain-framed messages were more effective mainly in specific patient populations (e.g., coronary artery disease) (40), whereas Liu et al. found age-dependent effects, with gain frames being more effective among older adults and loss frames among younger adults (41). The discrepancies may be better understood through an integration of classical theoretical models and contextual moderators. From the perspective of Prospect Theory, the “prevention-detection distinction” suggests that gain-framed messages are more persuasive for low-risk preventive behaviors, whereas loss-framed messages may be more effective for high-risk detection behaviors (42). Physical activity is typically conceptualized as a preventive behavior; however, its perceived risk may vary across populations (24, 43). Patient populations, particularly those with chronic conditions, may perceive PA as involving uncertainty or physical risk, thereby increasing risk salience and potentially attenuating the advantage of gain framing(27). In contrast, the general population may perceive PA as lower risk and more opportunity-oriented, making them more responsive to gain-framed appeals. Thus, differences in perceived behavioral risk across populations may partially explain the observed variation in framing effectiveness. Beyond risk perception, the Theory of Planned Behavior (TPB) provides an additional explanatory framework (44). Framed messages may influence PA behavior indirectly by shaping attitudes, PBC, and subjective norms. In younger adults, who often have greater physical capacity and fewer health constraints, gain-framed messages emphasizing positive outcomes may strengthen favorable attitudes and enhance perceived behavioral control, thereby increasing behavioral intention and subsequent action. In contrast, older adults may experience greater physical limitations or stronger habitual patterns, which could weaken the translation of framed attitudinal shifts into actual behavior (22). Moreover, if baseline self-efficacy or PBC is low, framing alone may be insufficient to produce meaningful behavioral change. Socio-cultural context may further moderate framing effects. Cultural norms regarding aging, health responsibility, and physical activity participation may influence how gain- or loss-framed message is interpreted (45). For example, in collectivist cultural settings, health behaviors may be evaluated in relation to family or social obligations, potentially altering sensitivity to gain versus loss emphasis (46). Additionally, gender differences may interact with framing valence. Some evidence suggests that men and women differ in risk perception, health motivation, and responsiveness to achievement-oriented versus threat-oriented messages. For instance, Li et al. reported that gain-framed messages were particularly effective in promoting PA among older men, possibly due to higher self-efficacy or greater discretionary time (22). Such findings imply that framing effects may operate through gender-specific motivational pathways. Finally, age-related cognitive and motivational differences may also play a role. According to socioemotional selectivity theory, older adults may prioritize emotionally meaningful or loss-avoidance goals, whereas younger adults may be more responsive to growth- and gain-oriented messages. These motivational shifts could influence the processing and persuasiveness of differently framed health communications (36, 47). Taken together, the effectiveness of gain- versus loss-framed messages in PA promotion appears to be shaped by an interaction between perceived behavioral risk, motivational and cognitive determinants, and contextual moderators such as age, gender, and socio-cultural background. However, given the limited number of subgroup analyses and the small sample sizes in existing trials, these interpretations remain tentative. Future research should explicitly test theoretically derived moderators, including perceived risk, self-efficacy, and socio-cultural variables, to clarify the mechanisms underlying framing effects in diverse populations.

Notably, previous research has demonstrated that the persuasiveness of the message framing is influenced by the kernel state of the message. For instance, within gain-framed messages, PA behavior is more effectively promoted by the obtain-desirable state than by the avoid-undesirable state, and vice versa (33, 48). However, only a limited number of studies included in this review explicitly examined the kernel state of the message frame. The other studies manipulated the kernel state in a message framing condition, such as gain framing messages with obtaining desirable or avoiding undesirable outcomes and loss framing messages with obtaining undesirable or avoiding desirable outcomes. Thus, we performed the analysis by classifying the gain- and non-loss framing messages into the gain-framed group and the loss- and non-gain framing messages into the loss-framed group. This would make it impossible to analyze the effect of the kernel state of the message frame on PA. Therefore, this point should be noted in future research, and the kernel state of gain-loss framing messages can be differentiated when conducting the research design to explore the effects of message framing on PA behavior in more depth, to find the optimal type of message framing that promotes PA.

### 4.1 Limitations

This study had several limitations. First, the number of eligible studies was limited, particularly for certain outcomes such as attitudes, and substantial heterogeneity was observed across studies. The small number of trials also restricted the robustness of subgroup analyses. Future research with larger sample sizes and more outcome-specific trials is needed to improve statistical power and enable more reliable subgroup investigations. Second, only physically inactive adults were included in this review. Therefore, the generalizability of our findings to physically active individuals or broader populations remains uncertain. Subsequent studies should examine framing effects across diverse activity levels and demographic groups to enhance external validity and clarify population-specific responses. Third, methodological variations across included studies may have contributed to heterogeneity. Assessment tools for PA attitudes, intentions, and behaviors were not standardized, with some studies relying on self-reported measures and others using objective assessments such as accelerometers. Differences in measurement instruments and outcome operationalization may have introduced variability and limited comparability across trials. In addition, the definition and reporting of message exposure dose were not consistently described, making it difficult to determine dose-response relationships or isolate the independent effects of framing valence. Future studies should adopt standardized measurement instruments and clearly define exposure characteristics to facilitate cross-study comparability and more precise evaluation of framing effects. Fourth, the limited availability of long-term follow-up data represents an important constraint. Although subgroup analyses based on follow-up duration were conducted, most included trials assessed immediate or short-term effects following a single or brief exposure, and only a small number reported outcomes at later time points. The uneven distribution of follow-up periods precluded more detailed meta-analyses across specific intervals (e.g., 2 weeks, 4weeks). As a result, the sustainability of gain- versus loss-framed messages on PA intentions and behaviors remains inconclusive. Future randomized controlled trials incorporating standardized measurement tools and longer-term follow-up assessments are needed to clarify potential temporal variations and durability of framing effects. Finally, due to heterogeneity in the moderators in these studies, we were not able to explore the interactions between other message characteristics and the gain-loss framework. Future studies could deeply explore the research mechanisms by which message framing affects individual PA to provide a theoretical basis for implementing message interventions.

## 5 Conclusions

Our meta-analysis did not demonstrate clear evidence of differences between gain-loss framed messages in promoting PA attitudes, intentions, and behaviors. Subgroup analyses suggested that gain-framed messages may show a potential advantage in promoting PA behaviors among young adults and individuals from the general population. However, these findings should be interpreted with caution given the limited number of studies and observed heterogeneity. These results indicate that gain-framed messages may be considered as a potentially promising approach for promoting PA in these populations, but further well-designed trials are needed to confirm their effectiveness and clarify underlying mechanisms.

## Data Availability

The original contributions presented in the study are included in the article/Supplementary material, further inquiries can be directed to the corresponding authors.
