# Supplementary material for: The effects of gain-loss framed message on physical activity attitudes, intentions, and behaviors in physically inactive adults: a systematic review and meta-analysis
Source: Front Public Health. 2026 Mar 16;14:1782478. doi: 10.3389/fpubh.2026.1782478 (PMC13033674; doi:10.3389/fpubh.2026.1782478)
Supplement: Supplementary file 1 [file Data_Sheet_1.docx]

Supplementary Material

# Search strategy

**PubMed**

| #1 | (((((((((((((message framing[Title/Abstract]) OR (message fram*[Title/Abstract])) OR (information framing[Title/Abstract])) OR (information fram*[Title/Abstract])) OR (gain-framed[Title/Abstract])) OR (loss-framed[Title/Abstract])) OR (gain fram*[Title/Abstract])) OR (loss fram*[Title/Abstract])) OR (positive frame[Title/Abstract])) OR (positive fram*[Title/Abstract])) OR (negative frame[Title/Abstract])) OR (negative fram*[Title/Abstract])) OR (goal fram*[Title/Abstract])) OR (framing effect*[Title/Abstract]) |
| --- | --- |
| #2 | ((((((((motor activity[MeSH Terms]) OR (aerobic exercise[MeSH Terms])) OR (exercise[MeSH Terms])) OR (sports[MeSH Terms])) OR (resistance training[MeSH Terms])) OR (Exercise Movement Techniques[MeSH Terms])) OR (Tai Ji[MeSH Terms])) OR (yoga[MeSH Terms])) OR (exercise therapy[MeSH Terms]) |
| #3 | ((((((((((((((((motor activity[Title/Abstract]) OR (aerobic exercise[Title/Abstract])) OR (exercise[Title/Abstract])) OR (aerobic exercis*[Title/Abstract])) OR (sport*[Title/Abstract])) OR (physical activity[Title/Abstract])) OR (physical activ*[Title/Abstract])) OR (physical exercise[Title/Abstract])) OR (physical exercis*[Title/Abstract])) OR (exercise training[Title/Abstract])) OR (exercise train*[Title/Abstract])) OR (exercise* prescription[Title/Abstract])) OR (exercise* therap*[Title/Abstract])) OR (resistance training[Title/Abstract])) OR (exercise movement techniques[Title/Abstract])) OR (Tai Ji[Title/Abstract])) OR (yoga[Title/Abstract]) |
| #4 | #2 OR #3 |
| #5 | (((attitude[MeSH Terms]) OR (intention[MeSH Terms])) OR (behavior[MeSH Terms])) OR (motivation[MeSH Terms]) |
| #6 | ((((((attitude[Title/Abstract]) OR (intention[Title/Abstract])) OR (behavior[Title/Abstract])) OR (motivation[Title/Abstract])) OR (behavio*[Title/Abstract])) OR (belief[Title/Abstract])) OR (belief*[Title/Abstract]) |
| #7 | #5 OR #6 |
| #8 | #1 AND #4 AND #7 |

**Embase**

| #1 | 'message framing':ti,ab,kw OR 'message fram*':ti,ab,kw OR 'information framing':ti,ab,kw OR 'information fram*':ti,ab,kw OR 'gain framed':ti,ab,kw OR 'loss framed':ti,ab,kw OR 'gain fram*':ti,ab,kw OR 'loss fram*':ti,ab,kw OR 'positive frame':ti,ab,kw OR 'positive fram*':ti,ab,kw OR 'negative frame':ti,ab,kw OR 'negative fram*':ti,ab,kw OR 'goal fram*':ti,ab,kw OR 'framing effect*':ti,ab,kw |
| --- | --- |
| #2 | 'motor activity'/exp OR 'aerobic exercise'/exp OR 'exercise'/exp OR 'sport'/exp OR 'resistance training'/exp OR 'kinesiotherapy'/exp OR 'tai chi'/exp OR 'yoga'/exp |
| #3 | 'motor activity':ti,ab,kw OR 'aerobic exercise':ti,ab,kw OR exercise:ti,ab,kw OR sport:ti,ab,kw OR 'resistance training':ti,ab,kw OR kinesiotherapy:ti,ab,kw OR 'tai chi':ti,ab,kw OR yoga:ti,ab,kw OR 'aerobic exercis*':ti,ab,kw OR sport*:ti,ab,kw OR 'physical activity':ti,ab,kw OR 'physical activ*':ti,ab,kw OR 'physical exercise':ti,ab,kw OR 'physical exercis*':ti,ab,kw OR 'exercise training':ti,ab,kw OR 'exercise train*':ti,ab,kw OR 'exercise* prescription':ti,ab,kw OR 'exercise* therap*':ti,ab,kw |
| #4 | #2 OR #3 |
| #5 | 'attitude'/exp OR 'behavior'/exp OR 'motivation'/exp |
| #6 | attitude:ti,ab,kw OR intention:ti,ab,kw OR behavior:ti,ab,kw OR motivation:ti,ab,kw OR behavio*:ti,ab,kw OR belief:ti,ab,kw OR belief*:ti,ab,kw |
| #7 | #5 OR #6 |
| #8 | #1 AND #4 AND #7 |

**CINAHL**

| S1 | TI message framing OR TI message fram* OR TI information framing OR TI information fram* OR TI gain-framed OR TI loss-framed OR TI gain fram* OR TI loss fram* OR TI positive frame OR TI positive fram* OR TI negative frame OR TI negative fram* OR TI message framing OR TI message fram* OR TI information framing OR TI information fram* OR TI gain-framed OR TI loss-framed OR TI gain fram* OR TI loss fram* OR TI positive frame OR TI positive fram* OR TI negative frame OR TI negative fram* OR TI goal fram* OR TI framing effect* |
| --- | --- |
| S2 | AB message framing OR AB message fram* OR AB information framing OR AB information fram* OR AB gain-framed OR AB loss-framed OR AB gain fram* OR AB loss fram* OR AB positive frame OR AB positive fram* OR AB negative frame OR AB negative fram* OR AB message framing OR AB message fram* OR AB information framing OR AB information fram* OR AB gain-framed OR AB loss-framed OR AB gain fram* OR AB loss fram* OR AB positive frame OR AB positive fram* OR AB negative frame OR AB negative fram* OR AB goal fram* OR AB framing effect* |
| S3 | S1 OR S2 |
| S4 | SU motor activity OR SU aerobic exercise OR SU exercise OR SU sports OR SU resistance training OR SU exercise movement techniques OR SU Tai Ji OR SU yoga OR SU exercise therapy |
| S5 | TI motor activity OR TI aerobic exercise OR TI exercise OR TI aerobic exercis* OR TI sport* OR TI physical activity OR TI physical activ* OR TI physical exercise OR TI physical exercis* OR TI exercise training OR TI exercise train* OR TI exercise* prescription OR TI exercise* therap* OR TI resistance training OR TI exercise movement techniques OR TI Tai Ji OR TI yoga |
| S6 | AB motor activity OR AB aerobic exercise OR AB exercise OR AB aerobic exercis* OR AB sport* OR AB physical activity OR AB physical activ* OR AB physical exercise OR AB physical exercis* OR AB exercise training OR AB exercise train* OR AB exercise* prescription OR AB exercise* therap* OR AB resistance training OR AB exercise movement techniques OR AB Tai Ji OR AB yoga |
| S7 | S4 OR S5 OR S6 |
| S8 | SU attitude OR SU intention OR SU behavior OR SU motivation |
| S9 | TI attitude OR TI intention OR TI behavior OR TI motivation OR TI behavio* OR TI belief OR TI belief* |
| S10 | AB attitude OR AB intention OR AB behavior OR AB motivation OR AB behavio* OR AB belief OR AB belief* |
| S11 | S8 OR S9 OR S10 |
| S12 | S3 AND S7 AND S11 |

**Cochrane Library**

| #1 | (message framing):ti,ab,kw OR (message framing):ti,ab,kw OR (information framing):ti,ab,kw OR (information fram*):ti,ab,kw OR (gain-framed):ti,ab,kw |
| --- | --- |
| #2 | (loss-framed):ti,ab,kw OR (gain fram*):ti,ab,kw OR (loss fram*):ti,ab,kw OR (positive frame):ti,ab,kw OR (positive fram*):ti,ab,kw |
| #3 | (positive fram*):ti,ab,kw OR (negative fram*):ti,ab,kw OR (goal fram*):ti,ab,kw OR (framing effect*):ti,ab,kw |
| #4 | #1 OR #2 OR #3 |
| #5 | motor activity |
| #6 | aerobic exercise |
| #7 | exercise |
| #8 | sports |
| #9 | resistance training |
| #10 | Tai Ji |
| #11 | yoga |
| #12 | exercise therapy |
| 13 | (motor activity):ti,ab,kw OR (aerobic exercise):ti,ab,kw OR (exercise):ti,ab,kw OR (aerobic exercis*):ti,ab,kw OR (sport*):ti,ab,kw |
| 14 | (physical activity):ti,ab,kw OR (physical activ*):ti,ab,kw OR (physical exercise):ti,ab,kw OR (physical exercis*):ti,ab,kw OR (exercise training):ti,ab,kw |
| 15 | (exercise train*):ti,ab,kw OR (exercise*):ti,ab,kw OR (exercise* therap*):ti,ab,kw OR (resistance training):ti,ab,kw OR (exercise movement techniques):ti,ab,kw |
| 16 | (Tai Ji):ti,ab,kw OR (yoga):ti,ab,kw |
| 17 | #5 OR #6 OR #7 OR #8 OR #9 OR #10 OR #11 OR #12 OR #13 OR #14 OR #15 OR #16 |
| 18 | attitude |
| 19 | intention |
| 20 | behavior |
| 21 | motivation |
| 22 | (attitude):ti,ab,kw OR (intention):ti,ab,kw OR (behavior):ti,ab,kw OR (motivation):ti,ab,kw OR (behavio*):ti,ab,kw |
| 23 | (belief):ti,ab,kw OR (belief*):ti,ab,kw |
| 24 | #18 OR #19 OR #20 OR #21 OR #22 |
| 25 | #4 AND #17 AND #24 |
| 26 | #25 AND Trails |

**Web of Science**

| #1 | (((TS=("message fram*" OR "information fram*" OR gain-fram* OR loss-fram* OR "positive fram*" OR "negative fram*" OR "goal fram*" OR "framing effect*")) OR TI=("message fram*" OR "information fram*" OR gain-fram* OR loss-fram* OR "positive fram*" OR "negative fram*" OR "goal fram*" OR "framing effect*")) OR AB=("message fram*" OR "information fram*" OR gain-fram* OR loss-fram* OR "positive fram*" OR "negative fram*" OR "goal fram*" OR "framing effect*")) |
| --- | --- |
| #2 | (((TS=(motor activit* OR aerobic exercise* OR exercise* OR sport* OR resistance training OR Exercise Movement Technique* OR yoga OR Taiji OR "exercise therap*")) OR TI=(motor activit* OR aerobic exercise* OR exercise* OR sport* OR resistance training OR Exercise Movement Technique* OR yoga OR Taiji OR "exercise therap*")) OR AB=(motor activit* OR aerobic exercise* OR exercise* OR sport* OR resistance training OR Exercise Movement Technique* OR yoga OR Taiji OR "exercise therap*")) |
| #3 | ((TS=(attitude* OR intention* OR behavior* OR motivation* )) OR TI=(attitude* OR intention* OR behavior* OR motivation* )) OR AB=(attitude* OR intention* OR behavior* OR motivation* ) |
| #4 | #1 AND #2 AND #3 |

**CNKI**

(SU %= '信息框架' OR SU %= '框架效应' OR SU %= '积极框架' OR SU %= '消极框架' OR SU %= '正面框架' OR SU %= '负面框架' OR SU %= '收益框架' OR SU %= '损失框架' OR SU %= '目标框架') AND (SU %= '运动' OR SU %= '身体活动' OR SU %= '锻炼' OR SU %= '运动康复' OR SU %= '运动疗法' OR SU %= '康复训练' OR SU %= '康复锻炼' OR SU %= '太极' OR SU %= '瑜伽') AND (SU %= '态度' OR SU %= '意愿' OR SU %= '行为' OR SU %= '动机' OR SU %= '信念')

**Wangfang Database**

主题:(信息框架 or 框架效应 or 积极框架 or 消极框架 or 正面框架 or 负面框架 or 收益框架 or 损失框架 or 目标框架) and 主题:(运动 or 身体活动 or 锻炼 or 运动康复 or 运动疗法 or 康复训练 or 太极 or 瑜伽) and 主题:(态度 or 意愿 or 行为 not 动机 or 信念)
