# Supplementary material for: The effects of gain-loss framed message on physical activity attitudes, intentions, and behaviors in physically inactive adults: a systematic review and meta-analysis
Source: Front Public Health. 2026 Mar 16;14:1782478. doi: 10.3389/fpubh.2026.1782478 (PMC13033674; doi:10.3389/fpubh.2026.1782478)
Supplement: Supplementary file 2 [file Data_Sheet_2.docx]

Supplementary Material

# Supplementary Figures and Tables

## Supplementary Table

**Table 1 The characteristics of studies.**

| **Study ID**  **&Country** | **Participant** | | | **Message framing design** | **Intervention times & Frequency** | **Research setting & Delivery channel** | **Follow-up time point( s)** | **Outcome & measure** |
| --- | --- | --- | --- | --- | --- | --- | --- | --- |
|  | **Population** | **Age & Female%** | **Sample size**  **(n=GF, LF)** |  |  |  |  |  |
| Latimer, et al. (2008) USA | Sedentary and healthy participants who engaged in MVPA no more than 2 days per week for more than 20 minutes per day | 47.40±12.03, 87% | 97 (50, 47) | Gain-, Loss-, Mixed frame | 3 times  (Once each for baseline, 1 week, 5 weeks) | Offline,  Print material | 2 weeks,  9 weeks | Intention, Behavior  （IPAQ） |
| Bassett, et al. (2013) Canada | Patients with SCI who did not meet the criteria engaging in 30 minutes of MVPA on at least 4 days per week | 45±12, 39.4% | 53 (27, 26) | Gain-, Loss-frame | Once | Online,  Electronic link | Immediately | Intention |
| **Study ID**  **&Country** | **Participant** | | | **Message framing design** | **Intervention times & Frequency** | **Research setting & Delivery channel** | **Follow-up time point( s)** | **Outcome & measure** |
|  | **Population** | **Age & Female%** | **Sample size**  **(n=GF, LF)** |  |  |  |  |  |
| de Bruijn, et al. (2014) Canada | Adults who did not engage in three bouts of MVPA per week, with each bout lasting at least 20 minutes | 32.38±13.22, 61.7% | 192 (90,102) | 2(Frame:gain/loss)×  2(kernel state:attained/  avoided) | Once | Online,  Leaflet | Immediately | Intention |
| Berenbaum, et al. (2014) Canada | Female college students who did not meet the recommended levels of ≥150 minutes per week of MVPA | 20.10±2.47, 100% | 60 (30, 30) | Gain-, Loss-frame | Once | Offline,  Digital print ads | Immediately, 1week | Attitude, Intention, Behavior  (IPAQ) |
| Kin-Kit, et al. (2014) China | Sedentary younger and older adults who did not meet the recommended levels of ≥150 minutes per week of MVPA | Younger:  22.31±3.04  Older: 71.66±7.48, 68% | 211 (107, 104) | Gain-, Loss-frame | Once | Offline,  Print material | 2 weeks | Behavior  (IPAQ,  Accelerometer) |
| **Study ID**  **&Country** | **Participant** | | | **Message framing design** | **Intervention times & Frequency** | **Research setting & Delivery channel** | **Follow-up time point( s)** | **Outcome & measure** |
|  | **Population** | **Age & Female%** | **Sample size**  **(n=GF, LF)** |  |  |  |  |  |
| Hirschey, et al. (2016) USA | Inactive CRC survivors who participated in < 150 minutes of MVPA per week | 64.82±11.61, 54% | 137 (72, 65) | Gain-, Loss-frame | Once | Offline,  Print material | 1 month, 12months | Attitude, Intention,  Behavior  (GLTEQ) |
| Lithopoulos, et al. (2017) Canada | Patients with multiple sclerosis who engaged in less than 30 minutes of MVPA twice per week | 41.57±9.58, 82.3% | 63 (35, 28) | 2 (Risk information: risk/no risk) ×  2 (Frame: gain/loss) | Four times (Once a day for 4 days) | Online,  Leaflet | Immediately, 4weeks | Intention, Behavior  (LTPAQ-SCI) |
| Kin-Kit, et al. (2017) China | Older adults with type 2 diabetes who participated in<150 minutes of PA in the previous week | 71.41±6.80, 52% | 211 (107, 104) | Gain-, Loss-frame | Once | Offline,  Print material | 2 weeks | Behavior  (Accelerometer) |

| **Study ID**  **&Country** | **Participant** | | | **Message framing design** | **Intervention times & Frequency** | **Research setting & Delivery channel** | **Follow-up time point( s)** | **Outcome & measure** |
| --- | --- | --- | --- | --- | --- | --- | --- | --- |
|  | **Population** | **Age & Female%** | **Sample size**  **(n=GF, LF)** |  |  |  |  |  |
| Kin-Kit, et al. (2021) China | College students who did not meet the recommended levels of ≥150 minutes per week of MVPA | 20.25±2.16, 60% | 147 (75, 72) | 2 (Frame: gain/loss) ×  2 (Endstate: positive/negative) | Once | Offline,  Print material | Immediately,  2 weeks | Attitude, Intention,  Behavior  (IPAQ) |
| Carfora, et al. (2022) Italy | Adults who engaged in <150 minutes of moderate-intensity PA per week or the equivalent of 75 minutes of vigorous-intensity PA | 18-65, 54.4% | 188 (95, 93) | Gain-, Non-loss, Non-gain, Loss- frame | Fourteen times (Once a day for 14 days) | Online,  APP | Immediately, 2weeks | Intention, Behavior  (Frequency of physical activity) |

Abbreviation: GF = Gain-framed group; LF = Loss-framed group; SCI: spinal cord injury; IPAQ = International Physical Activity Questionnaire = GLTEQ: Godin Leisure Time Exercise Questionnaire;

## LTPAQ-SCI = Leisure-time PA questionnaire for people with spinal cord injury; MAPV: moderate-to-vigorous physical activity.

## Supplementary Figures

| 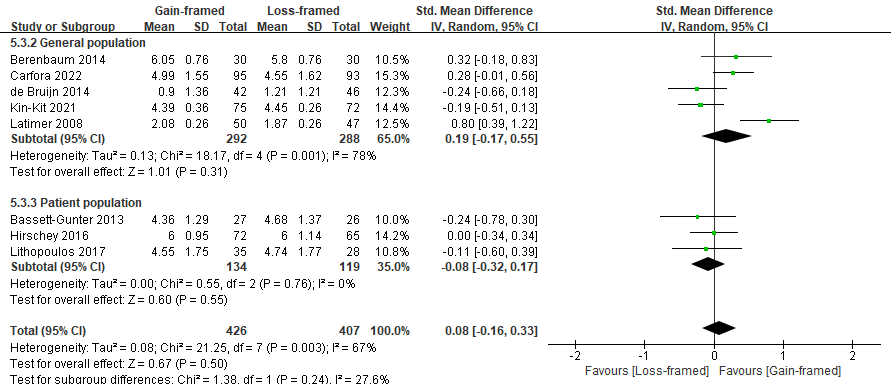 |
| --- |
| **Figure 1** Subgroup analyses of message framing on PA intention with different populations. |
| \| **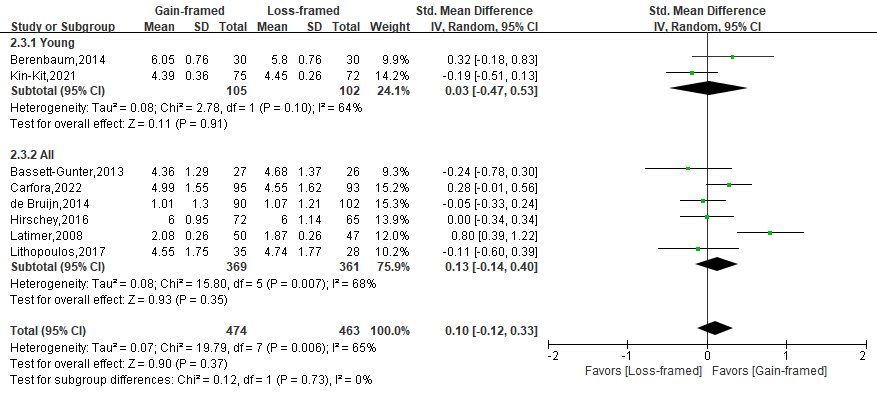** \| \| --- \| \| **Figure 2** Subgroup analyses of message framing on PA intention with different ages. \| |
| **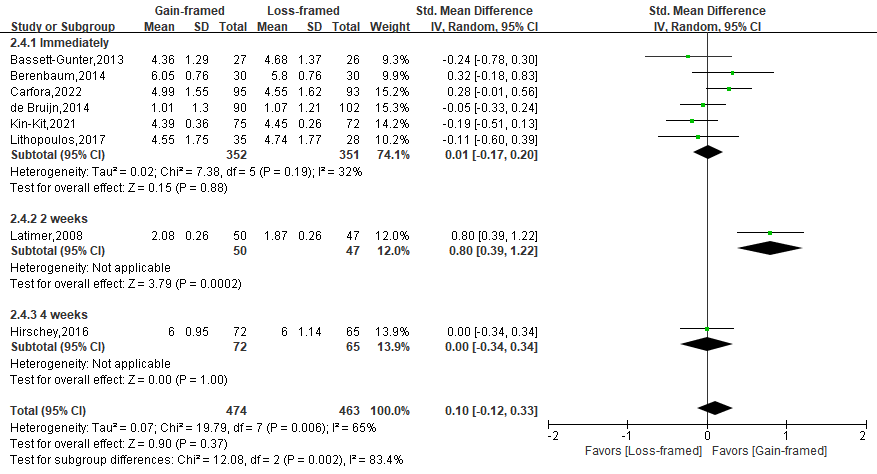** |
| **Figure 3** Subgroup analyses of message framing on PA intention with different follow-up durations. |

| **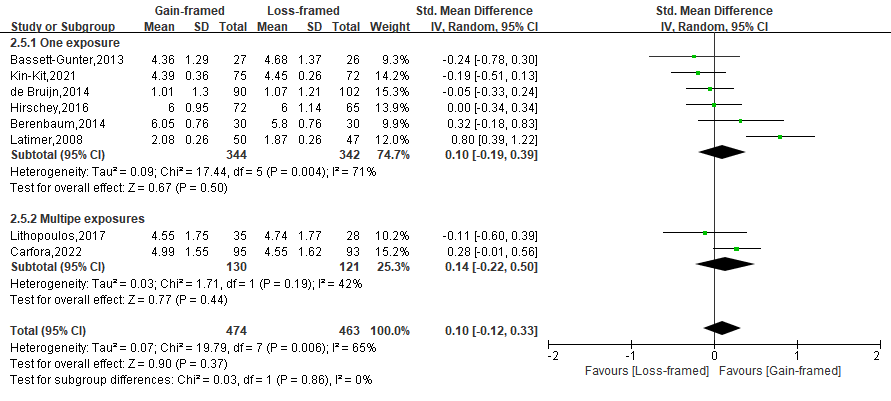** |
| --- |
| **Figure 4** Subgroup analyses of message framing on PA intention with different message exposure dosages. |
| **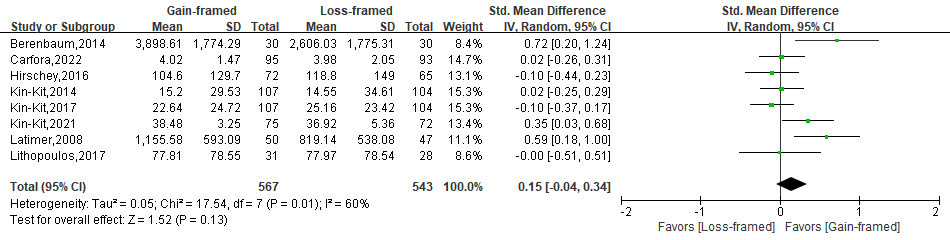** |
| **Figure 5** Forest plot of message framing on PA behavior. |

| **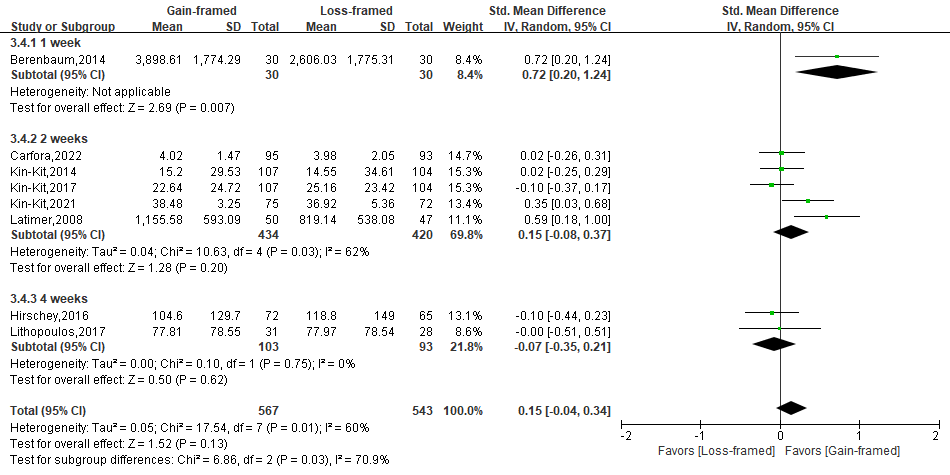** |
| --- |
| **Figure 6** Subgroup analyses of message framing on PA behavior with different follow-up durations. |

| **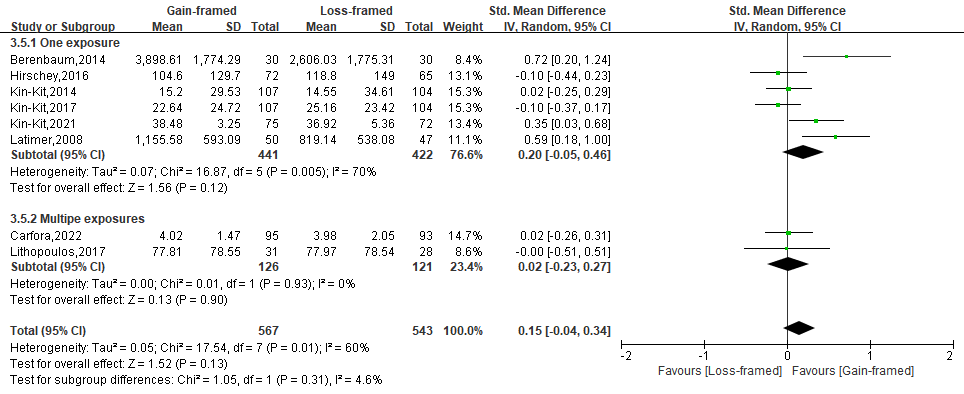** |
| --- |
| **Figure 7** Subgroup analyses of message framing on PA behavior with different message exposure dosages. |

| **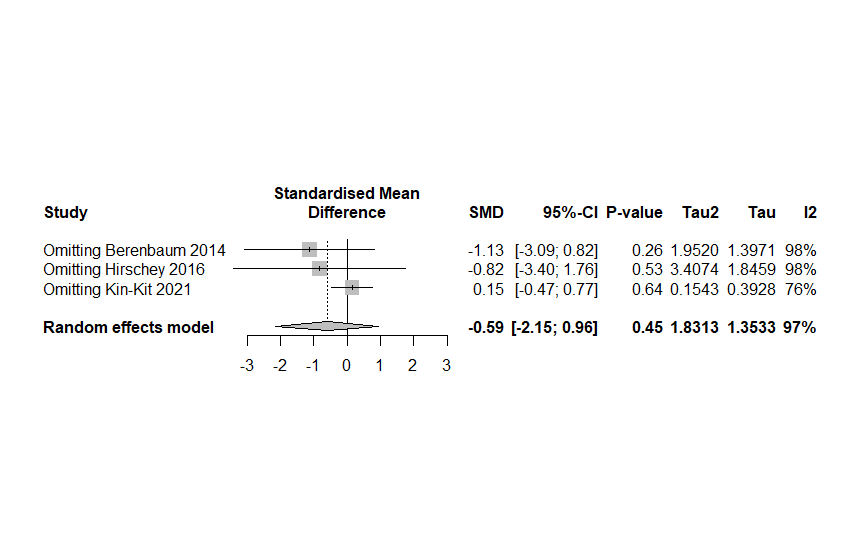** |
| --- |
| **Figure 8** Sensitivity analysis result of attitude. |
| **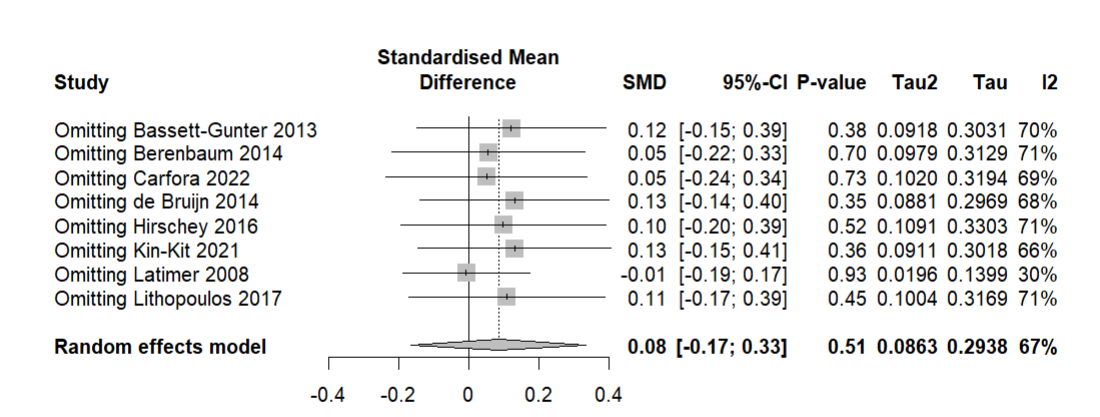** |
| **Figure 9** Sensitivity analysis result of intention. |

| **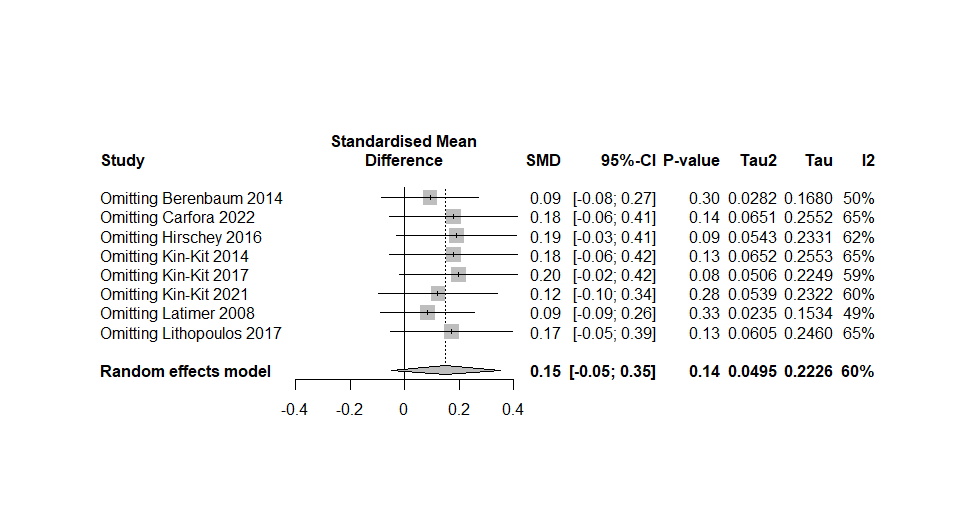** |
| --- |
| **Figure 10** Sensitivity analysis result of behavior. |
